# Anticancer and anti-angiogenic activities of mannooligosaccharides extracted from coconut meal on colorectal carcinoma cells *in vitro*

**DOI:** 10.1016/j.toxrep.2023.12.010

**Published:** 2023-12-30

**Authors:** Patthra Pason, Chakrit Tachaapaikoon, Waralee Suyama, Rattiya Waeonukul, Rong Shao, Molin Wongwattanakul, Temduang Limpaiboon, Chirapond Chonanant, Nipaporn Ngernyuang

**Affiliations:** a School of Bioresources and Technology, King Mongkut’s University of Technology Thonburi, Bangkok 10150, Thailand; b Pilot Plant Development and Training Institute, King Mongkut’s University of Technology Thonburi, Bangkok 10150, Thailand; c Shanghai Key Laboratory for Gallbladder Cancer-Related Gastroenterological Diseases, Xinhua Hospital, Shanghai Jiao Tong University School of Medicine, Shanghai 200089, China; d Centre for Research and Development of Medical Diagnostic Laboratories, Faculty of Associated Medical Science, Khon Kaen University, Khon Kaen 40002, Thailand; e Department of Medical Technology, Faculty of Allied Health Science, Burapha University, Chonburi 20131, Thailand; f Chulabhorn International College of Medicine, Thammasat University, Pathum Thani 12120, Thailand

**Keywords:** Mannooligosaccharides, Anti-cancer, Anti-angiogenesis

## Abstract

Colorectal carcinoma (CRC) is one of the most common malignancies, though there are no effective therapeutic regimens at present. This study aimed to investigate the inhibitory effects of mannooligosaccharides extracted from coconut meal (CMOSs) on the proliferation and migration of human colorectal cancer HCT116 cells *in vitro*. The results showed that CMOSs exhibited significant inhibitory activity against HCT116 cell proliferation in a concentration-dependent manner with less cytotoxic effects on the Vero normal cells. CMOSs displayed the ability to increase the activation of caspase-8, –9, and –3/7, as well as the generation of reactive oxygen species (ROS). Moreover, CMOSs suppressed HCT116 cell migration *in vitro*. Interestingly, treatment of human microvascular endothelial cells (HMVECs) with CMOSs resulted in the inhibition of cell proliferation, cell migration, and capillary-like tube formation, suggesting its anti-vascular angiogenesis. In summary, the results of this study indicate that CMOSs could be a valuable therapeutic candidate for CRC treatment.

## Highlights

• CMOSs exhibited inhibitory activity against HCT116 cell proliferation and migration.
• CMOSs induced cell cycle arrest in sub G0/G1 and increased cell apoptosis.
• There was an increase in caspase activity and ROS generation after CMOSs treatment.
• CMOSs suppressed the proliferation, migration, and tube formation of endothelial cells.

## Graphical Abstract

**Figure:**
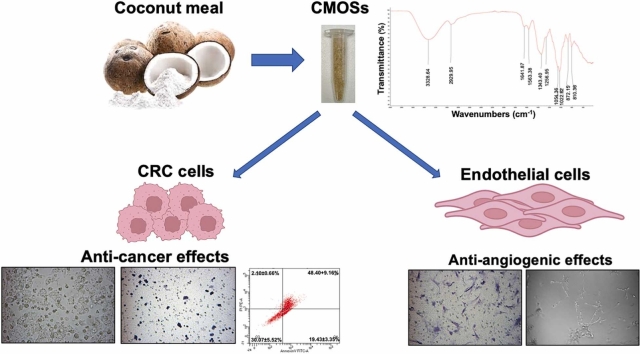

## 1 Introduction

Colorectal carcinoma (CRC), one of the most common forms of cancer, is the third leading cause of cancer-related death worldwide. At present, primary care for patients with CRC consists of surgical resection combined with adjuvant chemotherapy and/or targeted treatment based on clinicopathological features [1]. Chemotherapy for CRC involves both single-agent therapy (fluoropyrimidine 5-FU) and multiple-agent regimens such as oxaliplatin (OX), irinotecan (IRI), and capecitabine (CAP or XELODA or XEL). However, most of these anticancer drugs used in chemotherapy have shown a lack of efficacy with unexpected clinical outcomes such as systemic toxicity, low tumor-specific selectivity, and acquired drug resistance [2]. Therefore, there is an urgent need for the development of novel and effective anticancer drugs with minimal side effects for CRC treatment [3], [4]. Recently, natural plant products have received significant attention as the products commonly present in our food have shown therapeutic potential with low toxicity.

Coconut meal or copra meal is a by-product of coconut oil extraction and is generally regarded as agricultural waste with low nutritional value. Thailand is one of the largest producers of coconut and coconut milk worldwide, generating about 1.9 million tons of amounts of coconut meal each year [5]. For the above reasons, coconut meal is recognized as a promising feedstock for producing a variety of valuable products, particularly biofuel, and related products. However, coconut meal is most frequently used as fertilizer or as animal feed, with large quantities simply discarded [6]. The non-starch polysaccharides of coconut meal are comprised of galactomannan 61 %, mannan 26 %, and cellulose 13 % which can be used to produce valuable products [7]. Given the main content of galactomannan in coconut meal, it serves as a potential source to produce mannooligosaccharides (MOSs) [8], which are categorized as prebiotic non-digestible short-chain oligosaccharides consisting of 3–10 mannose residues [9]. MOS can be classified as alpha (α)- and beta (β)-MOS based on the glycosidic linkage present in the parent mannan polymer. The α-MOS is obtained from hydrolysis of α-1, 6- mannan present in the yeast cell wall, whereas β-MOS is derived from plant mannans linked by β-1, 4-glycosidic bonds [10]. MOSs are emerging prebiotics that have characteristic potential bio-active properties such as anti-cancer activities [8], [11], [12], [13], antioxidants [14], and anti-glycation activities [15]. Only a few studies have indicated that a variety of MOSs from different sources have demonstrated anti-cancer activities with no toxic responses, suggesting their potential as anti-cancer drug candidates [8], [11], [12], [13]. Ghosh and colleagues have demonstrated 50 % cell death of colon cancer cell line HT29 cells 24 h after treatment with 0.5 mg/mL MOS derived from copra meal [8]. MOS derived from palm kernel cake hydrolysis and coconut meal showed 74.19 % and 62.21 % cytotoxicity, respectively, against human colon adenocarcinoma cell line Caco-2 cell [13]. Moreover, galactomannan fractions from *Sesbania cannabina* inhibited the cell viability of several cancer cell lines such as HepG2, MCF-7, A549, and HeLa cells in a concentration-dependent manner [16]. However, little remains are known regarding the effects of coconut meal-derived mannooligosaccharides (CMOSs) on human CRC. The present study aims to demonstrate the biological effects of CMOSs on a human CRC cell line HCT116 and human microvascular endothelial cells (HMVECs). Results showing anti-CRC signatures may offer evidence of the therapeutic value of CMOSs in patients with CRC.

## 2 Experimental section

### 2.1 Extraction of CMOSs

The CMOSs used in the study were provided by the Pilot Plant Development and Training Institute, King Mongkut’s University of Technology Thonburi (Bangkok, Thailand) [17]. Briefly, *Bacillus tequilensis* (5.9 × 10^6^ CFU/mL) was cultured in a mineral salt medium (MS medium) containing 1 % w/v of coconut meal (pH 7.0). The reaction was conducted at 37 °C under continuous stirring for 24 h. Subsequently, the enzymatic reaction was stopped by boiling for 15 min. The sample was centrifuged at 7,104 xg for 10 min to remove any insoluble substances. Subsequently, the supernatant was collected and clarified by ultrafiltration. The CMOSs supernatant was freeze-dried and the resulting lyophilized products were used for further analysis. The lyophilized CMOSs were dissolved in sterile deionized (DI) water and kept at 4 °C for further experiments.

The chemical composition of CMOSs was analyzed following the National Renewable Energy Laboratory (NREL) (NREL protocol) [18]. The concentrations of oligosaccharide and sugar were measured by a high-performance liquid chromatograph (HPLC, Japan) with an Aminex-87 P column (Bio-Rad, USA) and detected by a refractive index detector (Shimadzu RID-10A, Japan) at 85 °C operated at 65 °C. Sulfuric acid (5 mM) at a flow rate of 0.6 mL/min was used for the mobile phase of both columns. The functional groups of CMOSs were investigated by Fourier-transform infrared spectroscopy (FTIR) using Nicolet iS50 FTIR spectrometers (Thermo Fisher Scientific). Spectra were recorded by averaging 64 scans, with a spectral resolution of 4 cm^-1^, covering a wavenumber range of 400–4000 cm^-1^.

### 2.2 Cell culture

Human CRC cell line HCT116 was purchased from ATCC (Manassas, VA). African green monkey kidney epithelial cell line Vero was used as the reference normal cells. HCT116 and Vero cells were cultured with Dulbecco's Modified Eagle's Medium-high glucose (DMEM-HG) supplemented with 10 % fetal bovine serum (FBS) and 1 % penicillin/streptomycin (P/S) (all from Life Technologies, Paisley, UK). HMVECs were grown in an endothelial basal medium (EBM-2) supplemented with 10 % FBS, 1 % P/S, 1 μg/mL hydrocortisone, and 10 ng/mL human epidermal growth factor (hEGF) (all from Lonza, Walkersville, MD, USA). All cells were cultured as a monolayer in a humidified atmosphere containing 5 % CO_2_ at 37 °C.

### 2.3 Cell viability assay

The effect of CMOSs on cellular viability was assessed by using a CellTiter 96® aqueous one-solution cell proliferation assay (MTS assay) (Promega, Madison, WI, USA). The HCT116 cells were seeded at a density of 5 × 10^3^ cells per well using 96-well plates in triplicate, then grown for confluency in 24 h. Cells were treated with different concentrations of CMOSs (1 to 40 mg/mL) or 5-Fluorouracil (5FU, 1 to 40 µg/mL) for 24 h. After treatment, the MTS solution was added to the wells, and plates were incubated for 1 h. Absorbance was detected at 490 nm using a microplate reader (Thermo Varioskan Flash Multi Detection microplate reader, Thermo Fisher Scientific). The percentage of viable cells was calculated by normalization with the untreated cells control. The IC_50_ value was calculated using a linear regression equation.

### 2.4 Morphological studies

The effect of CMOSs on cell morphology was examined using microscopic techniques. The HCT116 cells (1 ×10^5^ cells) were grown for 24 h and then incubated with CMOSs at IC_50_ 20 mg/mL. Morphological changes in the cells were observed at 200X magnification using a bright field inverted light microscope (Carl Zeiss Microscopy GmbH., Göttingen, Germany).

### 2.5 Clonogenic assay

The HCT116 cells were seeded at a density of 2 × 10^3^ cells per well in a 6-well plate for the colony formation assay. The cells were treated with 20 mg/mL of CMOSs for 7 days in triplicate. After treatment, the surviving cells were fixed with methanol and stained with a 0.5 % crystal violet solution at room temperature for 30 min. The number of colonies (>50 cells) was counted in six random fields under a light microscope. Calculation of the colony count percentage was performed relative to the untreated cells.

### 2.6 Cell cycle analysis

BD Cycletest™ Plus Reagent Kit (BD Biosciences, San Jose, CA, USA) was utilized to evaluate the cell cycle in the presence of CMOSs. Briefly, 2 × 10^6^ cells in a 6-well plate were treated with 20 mg/mL of CMOSs in triplicate for 24 h. Untreated cells were used as the control group. After treatment, cells were collected and the supernatant was removed. The cells were resuspended with solution A (trypsin buffer) and incubated for 10 min at room temperature. Afterward, the cells were incubated with solution B (trypsin inhibitor and RNase buffer) for 10 min at room temperature. Finally, the cells were incubated with cold solution C (propidium iodide (PI) solution) for 10 min in the dark on ice. The stained cells were detected by a Becton Dickinson flow cytometer (BD Biosciences). The cell cycle distribution was analyzed by BD FACSDiva software (BD Biosciences).

### 2.7 Annexin V-FITC apoptosis assay

To evaluate cell death induced by CMOSs, a FITC-annexinV apoptosis detection kit I (BD Biosciences) was utilized. Briefly, 2 × 10^6^ cells in a 6-well plate were treated with 20 mg/mL of CMOSs in triplicate for 24 h. Untreated cells were used as the control group. After treatment, the cells were collected and centrifuged at 507 xg for 3 min, after which the resulting pellets were washed twice with cold phosphate-buffered saline (PBS). Consequently, the cells were resuspended in a 1X binding buffer and stained with FITC-annexin V and PI in the dark at room temperature for 15 min. The stained cells were detected by a Becton Dickinson flow cytometer (BD Biosciences). The apoptotic rates were analyzed using BD FACSDiva software (BD Biosciences).

### 2.8 Caspases activity assay

Caspase-8, –9, and –3/7 activities were detected using a Caspase-Glo-8, –9, and –3/7 assay kit (Promega). HCT116 cells (5 × 10^3^ cells) were seeded into black 96-well plates in triplicate and treated with or without 20 mg/mL of CMOSs. After 24 h of treatment, the plates containing cells were removed from the incubator and placed at room temperature for 30 min. The Caspase-Glo solution was added to each well and then incubated at room temperature for 1 h. After incubation with the caspase solution, the luminescent signal was measured using a microplate reader. The percentage of caspase activity was calculated concerning untreated cells.

### 2.9 Detection of intracellular reactive oxygen species (ROS)

To evaluate the production of intracellular ROS, a non-fluorescent probe 2',7'-dichlorofluorescin diacetate (DCFH-DA; Sigma-Aldrich) assay was performed. Briefly, HCT116 cells (1 ×10^4^ cells/well) were seeded into 96 wells in triplicate and treated with or without 20 mg/mL of CMOSs. After treatment, cells were washed with PBS and stained with DCFH-DA at room temperature for 30 min. The cells were washed three times with PBS solution, and the fluorescence intensity was quantified. The fluorescent product was measured in triplicate by a microplate reader (Thermo Varioskan Flash Multi Detection microplate reader, Thermo Fisher Scientific) with an excitation wavelength of 485 nm and an emission wavelength of 537 nm. Data were presented as the mean percentage of ROS production compared to untreated cells set as 100 % ROS.

### 2.10 Transwell cell migration assay

The effect of CMOSs on cell migration was performed in a Transwell system (Costar Corning, Kennebunk, ME, USA). Briefly, a medium containing 10 % FBS with or without 20 mg/mL of CMOSs was added to the lower chamber, while HCT116 or HMVECs (2 ×10^5^ cells) were suspended in a serum-free medium and seeded into the upper chamber in triplicate. After 24 h of treatment, the upper insert well was gently removed and then washed three times with PBS. The non-migrated cells residing at the upper side of the filter were gently removed using a cotton swab applicator. Migrated cells that were attached to the underside of the filter were fixed with methanol and stained with a 0.5 % crystal violet solution at room temperature for 30 min. The number of migrating cells was counted in six random fields under a light microscope at 200X magnification. The total number of migrated cells in the untreated cells was designated as 100 %.

### 2.11 Capillary-like tube formation assay

The capillary-like structures formed by HMVECs on Matrigel (BD Biosciences) were evaluated. At least 30 min before the experiment, Matrigel was added to each well of a 96-well plate and allowed to polymerize at 37 °C. Subsequently, HMVECs (1 × 10^4^ cells) in the EBM-2 medium containing 10 % FBS with or without 20 mg/mL of CMOSs were seeded on a 96-well plate coated with Matrigel in triplicate. After 24 h of incubation, the number of capillary-like tube structure formations in each group was quantified in six random fields under an inverted phase-contrast light microscope at 200X magnification. The total tube number in the untreated cells was designated as 100 %.

### 2.12 Statistical analysis

Data were analyzed using SPSS version 20 software (IBM Corp., Armonk, NY, USA). Data are representative of three independent experiments and the values are expressed as mean ± standard deviation (SD). Comparisons between the groups were performed with the two-tailed Student’s *t*-test. Statistical significance was recognized at a value of *p* < 0.05.

## 3 Results and discussion

### 3.1 Characterization of CMOSs

**Fig. 1:**
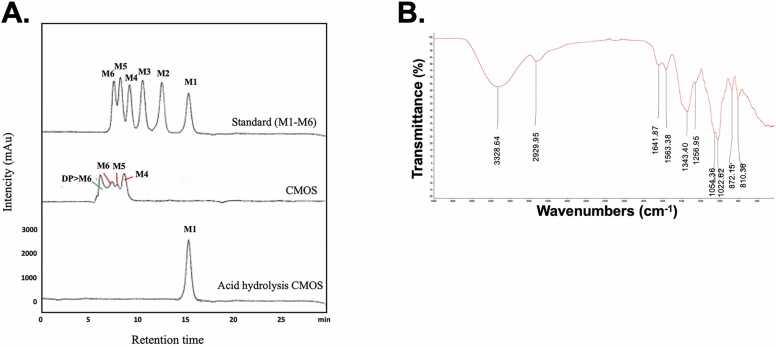
Characterization of CMOSs. (A) HPLC profiles of CMOSs and monosaccharide composition after complete acid hydrolysis compared to the standard mannose (M1) and mano-oligosaccharide (M2-M6). Manno-oligosaccharides (mannose (M1), mannobiose (M2), mannotriose (M3), mannotetraose (M4), mannopentaose (M5), and mannohexaose (M6) from Megazyme (Wicklow, Ireland)) were used as standards. (B) FTIR spectra of CMOSs.

The chemical composition of CMOSs was analyzed by HPLC. As shown in Fig. 1A, analysis of CMOS revealed that mannotetraose (M4) is a major product, following long-chain oligosaccharides (mannopentaose (M5) and mannohexaose (M6)). Complete acid hydrolysis CMOSs by sulfuric acid revealed mannose was detected as a single product. This result indicates that CMOSs are composed of mannose as a key monomer. The yield and product profile of MOS could vary depending on the source or type of the mannan hydrolyzed, the enzyme activity, and the hydrolysis conditions [19]. The FTIR analysis yielded prominent peak values representing the functional groups present in CMOSs, which are depicted in Fig. 1B alongside their corresponding wavenumbers and transmittance percentages. The absorption peak at approximately 3328.64 cm^-1^ can be attributed to the stretching vibrations of hydroxyl (O–H) groups, while the band at 2929.95 cm^-1^ corresponds to the C–H stretching of methyl groups [8], [20]. Additionally, the peaks at 1641.877 cm^-1^ and 1563.38 cm^-1^ were indicative of the bending vibrations of O–H groups. Notably, the characteristic absorption peak observed at 1343.40 cm^-1^ can be assigned to the variable angle vibrations of C-H bonds within the sugar ring. The absorption peaks of around 1256.95 cm^-1^, 1054.36 cm^-1^, and 1022.62 cm^-1^ indicate the stretching vibrations of C-O bonds within C-O-H or C-O-C groups, suggesting the presence of the pyranose ring structure in CMOSs [21]. Furthermore, the absorption bands at approximately 872.15 cm^-1^ and 810.36 cm^-1^ correspond to the variable-angle vibrations of C-H bonds, specifically related to the β-glycosidic bond in the compound. The results of this study were highly consistent with previously reported studies that showed the presence of O–H group stretching between 3200 cm^-1^ and 3400 cm^-1^ in MOS samples [8], [22], [23]. Indeed, Ghosh and colleagues previously observed the presence of C-H stretching in methyl and methylene groups at approximately 2930 cm^-1^ in coconut meal [8]. Moreover, the glycosidic linkage of the α-D-galactopyranose and β-D-mannopyranose units at 875 cm^-1^, 867 cm^-1^, and 873 cm^-1^ was found in the MOSs sample [22], which was identical in the presence of C-H stretching and glycosidic linkages in the CMOSs. Therefore, CMOSs represent the typical oligosaccharides containing pyranose rings. There are few studies demonstrating the anticancer of CMOSs. It is still unclear which components of CMOSs exert anticancer effects or mechanisms of action. However, several studies have reported that the anticancer activity of polysaccharides is also affected by the type and configuration of glycosidic bonds [24], [25], [26]. In general, the anticancer activity of polysaccharides with α-configuration has a lower anticancer activity, while that of polysaccharides with β-configuration possesses a higher activity [27].

### 3.2 Inhibition of cell proliferation and colony formation in HCT116 cells by CMOSs

**Fig. 2:**
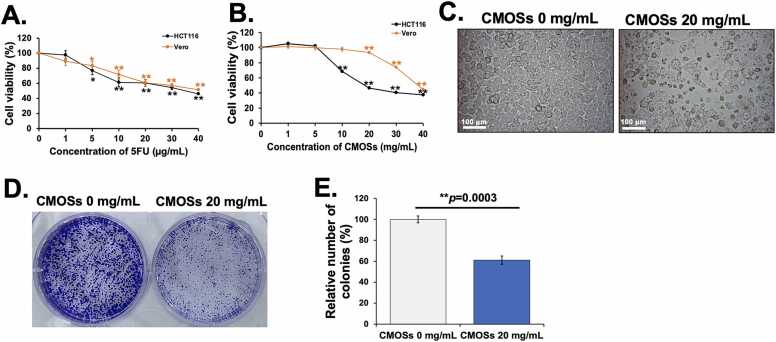
Effects of CMOSs on viability and colony formation. HCT116 and Vero cells were treated with different concentrations of (A) 5FU and (B) CMOSs for 24 h. The cytotoxic effects of 5FU and CMOSs on HCT cells and Vero cells were investigated using the MTS assay. (C) Typical images of the morphological alterations of untreated and treated HCT116 cells with CMOSs were obtained under 200X magnification. (D) A colony formation assay was performed to investigate the clone-forming ability of HCT116 cells treated with IC_50_ value of CMOSs (20 mg/mL) for 7 days. The cells in six-well culture plates were stained by crystal violet. (E) Quantitative data of colony percentages in untreated and treated HCT116 cells with CMOSs. Values are represented as mean±SD derived from three independent experiments. The ** *p* < 0.01 is considered statistically significant.

This study subsequently evaluated the antiproliferative effect of CMOSs on colorectal cancer cells *in vitro*. An immortalized normal cell line Vero and a human CRC cell line HCT116 were treated with various concentrations of CMOSs or 5FU for 24 h. 5FU, a chemotherapeutic drug used as a positive control, exhibited a dose-dependent manner with strong cytotoxicity in both HCT116 and Vero cells (Fig. 2A). CMOSs inhibited the proliferation of HCT116 cells in a dose-dependent manner (Fig. 2B). The IC_50_ level of CMOSs on HCT116 cells after treatment was 20 mg/mL, which was 2-fold lower than that of Vero cells (40 mg/mL), suggesting that CMOSs were less toxic to normal cells. Based on the results of the MTS assay, 20 mg/mL concentrations of CMOSs (IC_50_) were selected for further experiments.

To confirm the inhibitory effects, cells were treated with 20 mg/mL of CMOSs for 24 h, and the cell morphology was observed under light microscopy. The results in Fig. 2C demonstrate that CMOSs-treated cells were exfoliated, shrinking, and dead. In contrast, the untreated cells grew to be confluent with a healthy epithelial morphology. Moreover, the efficacy of CMOSs against HCT116 was evaluated after 7 days of CMOSs treatment in the colony assay. There was a significant decrease in colony formation by approximately 38.83 ± 3.95 % in CMOSs-treated cells when compared to untreated cells (Fig. 2D and E, *p* = 0.0003). The results suggest CMOSs could inhibit the growth and colony formation ability of HCT116 cells. A few studies have also implicated MOS in eliciting anticancer activity. The results in this study are in line with previous evidence reported by Ghosh and colleagues. They demonstrated that MOS derived from hydrolyzing defatted coconut meal blocked HT29 colorectal adenocarcinoma cell line proliferation after 24 h of treatment with IC_50_ 0.5 mg/mL [8]. Moreover, Juna and Kango found that treatment of the Caco-2 cell line with 1 mg/mL of coconut meal MOS resulted in a 62.21 % reduction in cell viability after 24 h [13]. In another study, 1 mg/mL of konjac gum oligosaccharides with M4 as the major content affected 30 % of cell death in hepatocellular carcinoma HepG2 cells via Bcl-2/Bax protein pathway [28]. The different IC_50_ in individual CRC cells indicate that the cytotoxicity of MOS is dependent on the targeted cell types and different products of MOS [29].

### 3.3 CMOSs induce cell cycle arrest in HCT116 cells

**Fig. 3:**
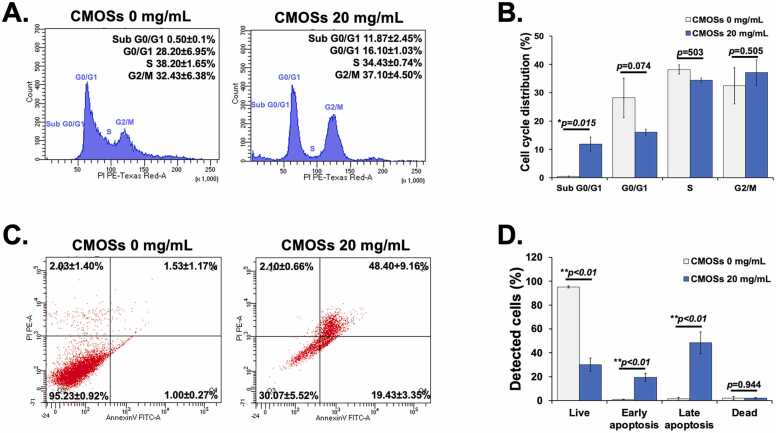
Effects of CMOSs on cell cycle progression and apoptosis in HCT116 cells using flow cytometry. (A) Representative flow cytometry histograms of PI fluorescence distributions of untreated and treated HCT116 cells with CMOSs. (B) Quantitative data of the percentage of cells in various phases of the cell cycle as analyzed by flow cytometry. (C) The apoptotic population of untreated and treated HCT116 cells with CMOSs was determined by Annexin-V assay. (D) Quantitative data of apoptosis percentages in untreated and CMOSs-treated cells. Values are represented as mean±SD derived from three independent experiments. The * *p* < 0.05 and * * *p* < 0.01 are considered statistically significant.

Cell cycle arrest is a common feature accompanying the inhibition of cancer cell proliferation [30]. Uncontrolled cell proliferation is a key biological feature that distinguishes cancer cells from normal somatic cells [31], in which the loss of cell cycle regulation is essential for cancer development [32]. The effect of CMOSs on the cell cycle was investigated on HCT116 cells. After the cells were treated with 20 mg/mL of CMOSs for 36 h, their distribution in different phases of the cell cycle was further investigated by flow cytometry analysis. The results showed that the HCT116 cells revealed a significant increase in the sub G0/G1 population (apoptotic cells) (37.23 ± 2.45 % versus 0.50 ± 0.10 %) (Fig. 3A and B), suggesting the increased toxicity of CMOSs. Moreover, the mean percentage of CMOSs-treated cells in the G2/M phase tended to increase compared to untreated cells (37.10 ± 4.50 % versus 32.43 ± 6.38 %, *p=*0.505), while the cells in G0/G1 and S phases decreased negligibly. These data suggest that the induction of apoptosis is a mechanism for growth inhibition of CMOSs. This finding was also consistent with a previous report that showed 1 mg/mL of apple oligosaccharide induces cell cycle arrest in the S phase, which correlated with the decreased expression of Cdk 2 and cyclin B1 in HT29 cells. Meanwhile, the low concentration of apple oligosaccharide (0.01 mg/mL and 0.1 mg/mL) did not induce cell cycle arrest in HT29 cells [33]. Moreover, neutral or acidic human milk oligosaccharides induced a concentration-dependent G2/M arrest in human intestinal cells, i.e. HT29, HIEC, and Caco-2 cells [34]. These results indicated that several oligosaccharides play an important role in attenuating colon cancer cell viability by inducing cell cycle arrest and cell apoptosis [33], [34]. The activation of cell cycle arrest might be an appropriate approach for cancer therapy [35]. Thus, CMOSs are a potential chemoprevention agent or anticancer agent and are worthy of further study.

### 3.4 Promotion of apoptosis by CMOSs through activation of extrinsic and intrinsic pathways in HCT116 cells

To address whether CMOSs inhibit apoptosis of HCT116 cells, flow cytometry using Annexin V and PI was determined. Annexin V is regularly used to identify apoptotic cells by binding explicitly to phosphatidylserine (PS), and PI is used to detect necrotic or late apoptotic cells [36]. As shown in Fig. 3C and D, the treatment of cells with 20 mg/mL of CMOSs led to 30.07 ± 5.52 % of live cells and 69.93 % of apoptotic cells compared with 95.23 ± 0.92 % of live cells and less than 5 % of apoptotic cells in untreated cells. This result was consistent with the cytotoxicity of CMOSs on HCT116 cells. A previous study has demonstrated that the flow cytometry result of 1 mg/mL apple polysaccharide induced apoptosis on HT29 cells [33].

**Fig. 4:**
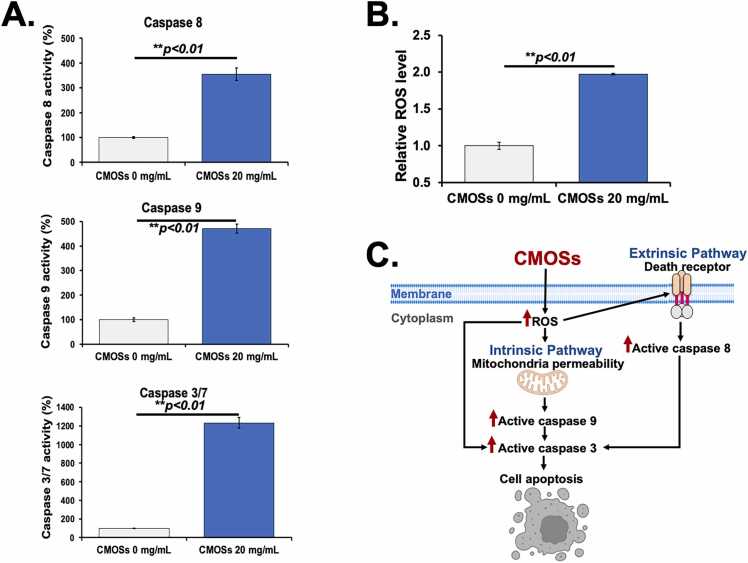
CMOSs induced caspase activities and ROS generation. (A) Activities of caspase –8, –9, and –3/7 were measured using luminescence assay. (B) DCFH-DA was measured for ROS levels using fluorescence in HCT116 cells after CMOSs treatment. Values are presented as mean±SD derived from three independent experiments. The * *p* < 0.05 and ** *p* < 0.01 are considered statistically significant. (C) Proposed mechanisms of CMOSs-induced apoptosis in HCT116 cells through caspases cascade activation and ROS generation.

It has been acknowledged that the extrinsic or death receptor pathway and the intrinsic or mitochondrial pathway participate in cell apoptosis, in which activation of caspase-8 mediates the extrinsic pathway, while caspase-9 activation initiates the intrinsic pathway [37]. To investigate if these pathways mediate CMOSs-initiated apoptosis in HCT116 cells, we evaluated the caspase activities by measuring specific luminescent products induced by caspase-8 (extrinsic pathway), caspase-9 (intrinsic pathway), and activation of executor caspase-3/7 (a downstream effector of both pathways). As shown in Fig. 4A, the CMOSs-treated cells (20 mg/mL) significantly enhanced the activities of caspase-8, caspase-9, and caspases-3/7 compared to untreated cells (*p* < 0.01). These findings suggested that CMOSs-induced apoptosis is associated with both intrinsic and extrinsic pathways. The results in this study highly agreed with the study by Ma et al., which demonstrated that homogeneous polysaccharides (HPS) extracted from Hawthorn can induce cell cycle arrest and regulate caspase-8, –9, and –3-mediated both intrinsic and extrinsic apoptotic pathways in HCT116 cells [38]. In addition, the fucose-containing sulfated polysaccharides (FCSPs) from *Sargassum henslowianum* and *Fucus vesiculosus* also showed the activated caspase-3 in the melanoma cells treated with FCSPs [39]. In context with other findings, our results indicate that the CMOSs induce apoptosis through both extrinsic and intrinsic pathways.

### 3.5 Increase of intracellular ROS production in HCT116 cells by CMOSs

Several studies have demonstrated that ROS generation is associated with the activation of both intrinsic and extrinsic pathways of apoptosis and is induced by numerous anti-cancer compounds [40], [41]. Thus, the current study hypothesized that CMOSs promoted ROS levels, which accounted for cell apoptosis. To test this hypothesis, the intracellular ROS level was measured using the ROS-sensitive dye DCHF-DA which can represent ROS regeneration. Fig. 4B shows that treatment of HCT116 cells with CMOSs at 20 mg/mL for 24 h resulted in a dramatic increase in the mean DCF fluorescence 1.97-fold more than that in the untreated cells (*p* < 0.01). This result indicates that CMOSs could induce intracellular ROS generation in HCT116 cells.

ROS can mediate apoptosis by regulating the expression of various pro-apoptotic proteins such as caspases [42]. In the extrinsic apoptosis pathway, ROS activates death receptors such as TNF-R1, TRAIL-R1/2, and FasR, which leads to the activation of initiator caspase-8 and the subsequent activation of caspase-3/7. In addition, ROS can also regulate apoptosis by directly affecting the pro- and anti-apoptotic proteins of the extrinsic pathway [40]. In the intrinsic apoptosis pathway (mitochondrial pathway), ROS induces mitochondrial membrane depolarization, resulting in the release of cytochrome c (Cyt c) into the cytosol. Cyt c can develop an apoptosome complex with procaspase-9, leading to caspase-9 activation. Caspase-9 then activates effector caspase-3/7, ultimately leading to the cleavage of cellular proteins and cell death [40]. Collectively, this study showed that the ROS triggered the progression of apoptosis through activation of both the caspase-9-independent mitochondrial pathway and caspase-8 death receptor pathways in CMOSs-treated HCT116 cells (Fig. 4C).

### 3.6 Inhibition of HCT116 cell migration by CMOSs

**Fig. 5:**
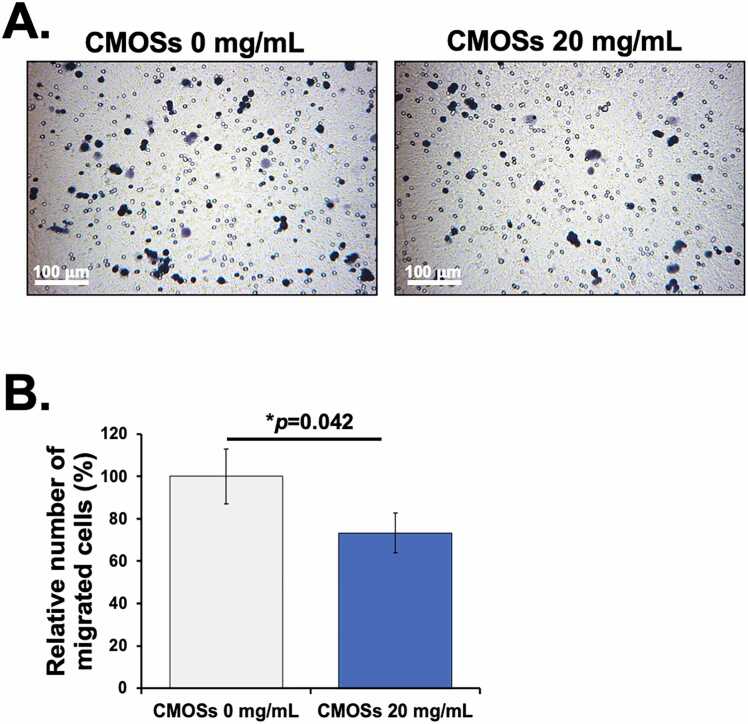
Inhibition of HCT116 migration by CMOSs. HCT116 was treated with CMOSs for 24 h, after which cell migration was evaluated using Transwell migration assay. (A) Representative results showed the effect of CMOSs on cell migration. (B) Quantitative data showed a significant decrease of migrating cells treated with CMOSs compared to untreated control. Values are presented as mean±SD derived from three independent experiments. The *p < 0.05 is considered statistically significant.

Cancer cell migration plays a vital role in cancer metastasis, which is an adverse prognostic factor contributing to CRC-related mortality [43]. To investigate whether CMOSs could inhibit the migration of HCT116 cells, the effect of CMOSs on cell motility was examined using a Transwell migration assay. As shown in Fig. 5, HCT116 cells treated with CMOSs significantly suppressed the migration of HCT116 cells by 59.53 % compared with untreated cells. These results indicate that CMOSs possess the ability to inhibit the migration of CRC. A previous study demonstrated *in vitro* that the spreading of B16 cells on the basement membrane depends on the N-linked high mannose carbohydrate structure [44]. Lui et al. investigated that the MOS had the effects of preventing melanoma cells from experimental metastasis to and out of the liver and prolonging the survival time of the Balb/c mouse [45].

### 3.7 Anti-angiogenic effects of CMOSs on endothelial cells

**Fig. 6:**
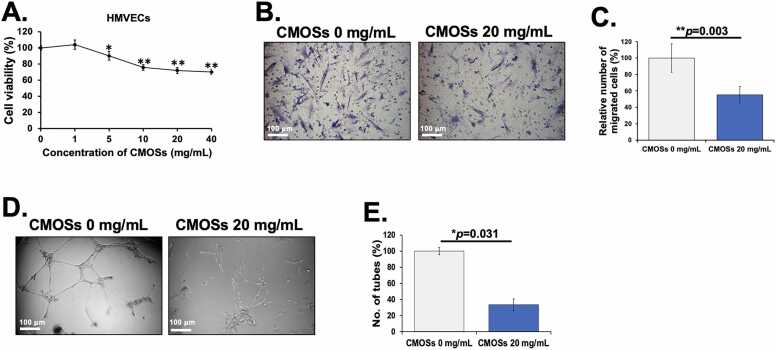
Anti-angiogenic effects of CMOSs on endothelial cells. (A) The number of viable cells after treatment was calculated relative to untreated cells. (B) Representative images showed the effect of CMOSs on the migration of HMVECs. (C) The number of HMVECs migrated through the membrane was quantified. (D) Representative images showed the effect of CMOSs on the capillary-like tube formation of HMVECs. (E) The number of capillary-like tube networks was quantified. Values are presented as mean±SD derived from three independent experiments. The * *p* < 0.05 and ** *p* < 0.01 are considered statistically significant.

Angiogenesis, the biological process of new blood vessel formation, plays an important role in tumor growth, maintenance, invasion, and metastasis. Increased tumor angiogenesis is associated with metastasis status and poor prognosis in CRC patients [46]. Therefore, anti-angiogenesis therapy has become an important therapeutic strategy in CRC [47]. To address whether the CMOSs inhibit tumor angiogenesis, we engaged the assays for the proliferation, migration, and capillary-like tube formation of HMVECs. To demonstrate the anti-angiogenic activity of CMOSs *in vitro*, their antiproliferative effect on HMVECs was evaluated using the MTS assay. As shown in Fig. 6A, the CMOSs significantly inhibited the proliferation of HMVECs in a dose-dependent manner [48]. Subsequently, a migration assay of HMVECs was performed using the Transwell assay. CMOSs significantly reduced the migration of HMVECs by 44.76 ± 10.08 % of the untreated cells (Fig. 6B and C). Finally, endothelial network formation was examined by Matrigel assay. As shown in Fig. 6D and E, the number of tubular structures was significantly decreased by 66.67 ± 1.53 % in the presence of 20 mg/mL of CMOSs. Thus, these results strongly suggest that CMOSs possess anti-angiogenic ability *in vitro*. There is only one report on MOSs having anti-tumorigenic potential where sulfonated MOSs from *Pichia pastoris* inhibited angiogenesis resulting in delayed tumor cell invasion and tumorigenesis [49]. Therefore, the investigations are essential to discover the potential of MOSs for delaying tumorigenesis.

On the whole, this study has described, for the first time, that CMOSs function to inhibit tumor cell proliferation and endothelial cell proliferation, migration, and capillary-like tube formation, but induce tumor cell cycle arrest and apoptosis. This study has provided key evidence to suggest that CMOSs may serve as a powerful agent to block CRC development.

## 4 Conclusion

**Fig. 7:**
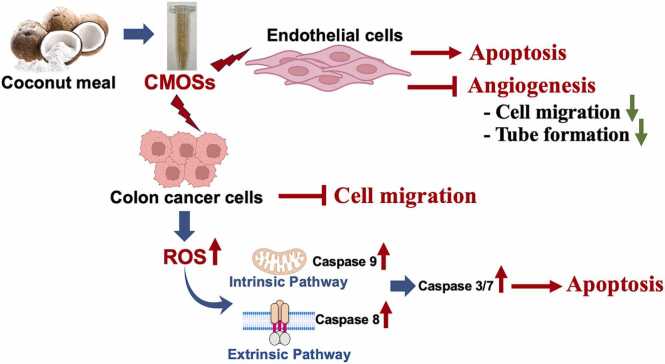
Schematic diagram summarizing the effect of CMOSs.

The present study demonstrated that CMOSs could inhibit tumor growth but induce cell cycle arrest and apoptosis by activating caspase-8, –9, and –3/7, and ROS generation through both intrinsic and extrinsic mechanisms in human colon cancer cell line HCT116. CMOSs also suppress the angiogenesis process including the inhibition of cell proliferation, cell migration, and capillary-like tube formation on HMVECs. In the next step, animal models should be taken into account to validate their therapeutic efficacy. To the best of the authors’ knowledge, this is the first time that it has been demonstrated that the CMOSs exert dual action in anticancer and anti-angiogenic activities (Fig. 7). Therefore, CMOSs comprise a promising option for CRC therapeutic approaches.

## CRediT authorship contribution statement

**Patthra Pason:** Methodology, Investigation, Validation, Writing- Reviewing and Editing. **Chakrit Tachaapaikoon:** Methodology, Formal analysis. **Waralee Suyama:** Methodology. **Rattiya Waeonukul:** Methodology. **Rong Shao:** Visualization. **Molin Wongwattanakul:** Formal analysis. **Temduang Limpaiboon:** Resources. **Chirapond Chonanant:** Formal analysis. **Nipaporn Ngernyuang:** Conceptualization, Methodology, Investigation, Project administration , Writing- Reviewing and Editing.

## Declaration of Competing Interest

The authors declare that they have no known competing financial interests or personal relationships that could have appeared to influence the work reported in this paper.

## Data Availability

No data was used for the research described in the article.
